# Smoking Bans in Psychiatric Units: An Issue of Medical Ethics

**DOI:** 10.3389/fpsyt.2019.00134

**Published:** 2019-03-20

**Authors:** Eleanor R. Woodward, Robyn Richmond

**Affiliations:** School of Public Health and Community Medicine, University of New South Wales, Sydney, NSW, Australia

**Keywords:** smoking, psychiatric units, ethics, smoke-free policies, tobacco control

## Introduction

Smoking and smoking-related illnesses are overrepresented among people with mental illness, contributing substantially to the reduced life expectancy observed in this group (1). Creating totally smoke-free inpatient psychiatric facilities is a goal that hospitals and policy makers are increasingly supporting, with a goal of improving health outcomes of both patients and staff (2).

The unique situation of many psychiatric patients in being unable to leave hospital grounds, however, means smoking bans remain a contentious issue in these settings (3, 4). Debates around patient choice, harm avoidance and the health service's moral imperative to promote well-being persist, leading to wide variations in the application of smoking bans across facilities (3, 5–7). While most psychiatric units do not allow smoking indoors, many only have partial smoke-free policies in place. This enables psychiatric patients to smoke outdoors on hospital grounds, often despite total smoking bans (indoor and outdoor) being in place for all other patients (8, 9).

This article evaluates the debate using a framework of the four basic principles of medical ethics—autonomy, justice, beneficence, and non-maleficence (Table 1). Within the context of each principle, arguments for and against total smoking bans in psychiatric facilities are evaluated, and evidence to support the most ethical way forward is discussed.

**Table 1:** The basic principles of medical ethics, applied to the major arguments for and against inpatient psychiatric unit smoking bans.

**Ethical principle**	**Arguments against smoking bans**	**Arguments for smoking bans**	**The most ethical way forward?**
Autonomy	Self-determination and paternalism: it is a patient's right to choose to smoke	Smoking is an “addiction,” not a lifestyle echoice	Addiction support strategies
Justice	Bans present an unfair disadvantage on psychiatric patients, given higher rates of smoking in this group	As a disproportionately burdened group, more must be done to address smoking among people with mental illness	Actively identify and support those with mental illness to quit smoking and remain smoke-free
Beneficence	“Self-medication” hypothesis: smoking as a behavioral and self-regulatory tool	Quitting smoking improves the health of patients/staff	Provide other, healthier self-regulatory measures
Non-maleficence	“Harm-minimization” approach: allowing smoking is favorable to the fallout from smoking bans	Smoking directly harms physical and mental health, and only total smoking bans reduce SHS to safe levels	Identify and manage the perceived potential fallout from smoking bans

## Autonomy

A key argument of those opposing total smoking bans is that such bans impose on patients' rights to self-determination, and therefore encroach on moral autonomy (4). Moreover, some argue that depriving patients of their ability to make decisions relating to smoking is a paternalistic approach to health, directly contradicting a recovery-focused approach to psychiatric care (4).

The issue of autonomy becomes more complex when smoking is viewed as an addiction, rather than a “choice.” Grounded in the historical and cultural milieu surrounding tobacco use, smoking is often considered at the “choice” end of the spectrum (3). Nicotine dependence is well-established, however, as a form of drug addiction, and treating it as a choice deviates significantly from the approach taken to other addictions (10). One may consider alcohol by way of comparison. A similarly legal substance with known addictive and harmful qualities, there is general consensus that alcohol should be completely prohibited in inpatient mental health facilities (3). When considered from this perspective, it can be argued that the obligation of healthcare workers to avoid enabling addictive behaviors becomes the priority (10). Furthermore, the legal status of smoking as a fundamental “right” has overturned in judicial proceedings in several counties (11, 12). This is supported by the viewpoint that allowing patients to smoke does not meet the threshold of a health officer's duty to provide competent care (10). Instead, the physician should endeavor to support patients in overcoming harmful addictions with therapies such as substitution pharmacotherapy and behavioral counseling. With robust evidence for the potential gains from these therapies in this population, this approach arguably takes ethical precedence over granting permission for patients to smoke in psychiatric units (13, 14).

## Justice

Health promotion interventions that unfairly burden certain groups risk violating the ethical cornerstone of justice (15). Those in opposition of smoking bans argue that comprehensive bans present an unfair disadvantage for psychiatric patients, given the substantially higher rates of smoking in this group (16). Others view denying mental health patients of cigarettes while they are acutely unwell as unfair and inhumane, particularly when many other liberties are already restricted (3, 4).

Conversely, proponents of total smoking bans view justice from the angle of health inequalities (17). From this perspective, it would be unfair to implicitly or explicitly enable behaviors that perpetuate the health and social inequities related to smoking. Moreover, from a proportionate universalism approach to justice, applying a comprehensive smoking ban on hospital grounds would be seen merely as a starting point. In order to fully address inequities related to tobacco use, additional evidence-based strategies should be implemented to support reductions for those most at risk of tobacco-related harm. A fair and humane approach to reducing smoking among this group must be fair in both the application of the ban as well as the proportionate supply of supports (17). Specific exemptions for psychiatric patients to smoke on hospital grounds would directly contradict this approach.

## Beneficence

The reasons people smoke are multifold and varied. Opponents of smoking bans argue that applying comprehensive smoke-free policies is a rationalistic approach that neglects to address the complexity of tobacco use in this population (16). A widespread belief held by many staff and patients is that tobacco smoking is helpful in the management of psychiatric disorders (3). Smoking has also been touted as a tool for stress relief, pleasure and socialization for patients (18, 19). These beliefs form part of the “self-medication” hypothesis, which proposes that people with mental illness smoke cigarettes to self-regulate psychiatric symptoms and psychosocial circumstances (20). With this hypothesis in mind, some question whether the intended outcome of smoke-free policies (reduced long-term smoking) justifies the means (depriving patients of a tool for self-regulation) (21). From this perspective, banning patients from smoking may violate the physicians' moral imperative to favor the well-being and interest of the patient—critical to the upholding of beneficence (22).

Significant uncertainty exists, however, in the true reasons for high rates of smoking among people with mental illness (23). A recent Cochrane Review on the use of nicotine for symptoms of schizophrenia reported no association, directly contradicting the self-medication hypothesis (24). “Smoking culture” in psychiatric care settings has instead frequently been reported as a key driver behind high smoking rates among both patients and staff (25). A lack of opportunities for socialization, entertainment, and stress alleviation contribute to this culture, which sees smoking embedded as part of daily life on the unit (26–28). Moving forward, the most ethical approach is to address smoking culture in psychiatric units by providing healthy alternatives to entertainment, socialization, and coping with stress. For example, there is evidence that diversional therapy and structured group activities can provide opportunities for socialization and promote relaxation while reducing boredom in inpatient units (26, 29). Given the evidence for the important gains of smoking cessation, such as reduced mortality and improved physical and mental health, it is difficult to justify allowing smoking on the grounds of beneficence (30). The goal should instead be to provide holistic care that tackles smoking culture within psychiatric units by providing alternative options for cognitive stimulation, relaxation, and socialization.

## Non-maleficence

The health worker's imperative to do no harm is a cornerstone of ethical practice. The potential harms to patients from smoking bans form a key thread of the argument against smoking bans (3). It has been suggested that smoking should be permitted in order to avoid consequences of nicotine withdrawal such as worsening of acute psychiatric, aggression and disruption among patients (17). Opponents of smoke-free policies also raise concerns that smoking bans may lead to patients absconding or deter patients from presenting to psychiatric facilities (3, 4). On these grounds, it has been argued that smoking be allowed in inpatient psychiatric facilities as an approach to harm-minimization (4).

Despite many studies demonstrating no increased aggression or disruption following implementation of smoking bans in psychiatric facilities, fear of aggressive backlash presents a major barrier to establishing smoking bans (3, 8, 31, 32). These concerns stem from the historical use of cigarettes as behavioral management tools and concerns of unmanageable withdrawal symptoms (3, 7, 26). As discussed previously, however, smoking is a well-established form of drug addiction and utilizing cigarettes as a management tool becomes ethically problematic under this light. Protecting patients from nicotine withdrawal is certainly necessary in avoiding the symptoms of withdrawal that can lead to disruptions in mood and behavior, and on these grounds some call for partial smoking bans (33). Evidence suggests that partial smoking bans —that is, bans that enable patients to smoke intermittently while outside on hospital grounds—actually place patients in chronic nicotine withdrawal, however, rather than relieving withdrawal symptoms (3, 34, 35). Furthermore, intermittent access to cigarettes creates conditions for physiological reinforcement, which strengthens the cycle of nicotine addiction (36). On the other hand, evidence suggests withdrawal symptoms can be managed in this group with substitution therapies such as nicotine replacement therapy (3). Together, this suggests nicotine withdrawal can and should be managed in conjunction with smoke free policies, to adequately protect patients in inpatient units from withdrawal symptoms.

In contemplating non-maleficence, the aforementioned risks of smoking bans must be considered against the robust evidence for the significant consequences of tobacco use among people with mental illness. Smoking is the leading cause of preventable death in this population, and is associated with significant morbidity, psychosocial risks and economic losses (9). The harms of cigarette-related disease in this group arguably outweigh the potential for harms resulting from smoking bans. This is particularly pertinent, given the potential fallout from smoking bans are much more easily managed than the long-term, often irreversible physical harms caused by tobacco use. Furthermore, quitting smoking has repeatedly been linked to improved mental well-being (30, 37, 38). As the overarching objective of admission to psychiatric facilities is to support mental health, this adds an important element to the argument for smoke-free policies in these settings.

Moreover, consideration should be given to the well-established harms of second-hand smoke exposure to visitors, staff and other patients in facilities without comprehensive smoke-free policies in place (39, 40). Research has shown that only total smoking bans protect against harmful levels of second-hand smoke exposure in psychiatric facilities (41). Alarmingly, other types of bans (partial bans or no bans) have been linked with up to a 15% reduction in life expectancy, according to the World Health Organization air quality guidelines (41). This leaves little room for debate in developing policies that align with the principle of non-maleficence.

## Conclusion

Moving forward, the focus should be on a holistic, biopsychosocial approach to health. The factors that contribute to the overwhelmingly poor health and social outcome among the psychiatric population must be proactively addressed. Addiction disorders contributing directly to disparities, for which safe cessation support strategies are well-established, must not be enabled—either implicitly or explicitly. Hospitalization provides a unique opportunity to break the cycle of addiction for psychiatric patients and, given the devastating effects of smoking on this already significantly disadvantaged group, concerted efforts to reduce smoking seem to be the only ethical way forward.

## Author Contributions

EW contributed conception of the work, reviewing the literature, and writing the first draft. RR contributed by critically revising the work for important intellectual content. All authors contributed to the final manuscript revision, read, and approved the submitted version.

### Conflict of Interest Statement

The authors declare that the research was conducted in the absence of any commercial or financial relationships that could be construed as a potential conflict of interest.

## References

[1] Lawrence D Hancock KJ Kisely S. The gap in life expectancy from preventable physical illness in psychiatric patients in Western Australia: retrospective analysis of population based registers. Br Med J. (2013) 346:f2539. 10.1136/bmj.f2539 23694688 PMC3660620

[2] Stockings E Bowman J Baker A Terry M Clancy R Wye P. Impact of a postdischarge smoking cessation intervention for smokers admitted to an inpatient psychiatric facility: a randomized controlled trial. Nicotine Tobacco Res. (2014) 16:1417–28. 10.1093/ntr/ntu097 24939916

[3] Lawn S Campion J. Achieving smoke-free mental health services: lessons from the past decade of implementation research. Int J Environ Res Public Health. (2013) 10:4224–44. 10.3390/ijerph10094224 24025397 PMC3799524

[4] Arnott D Wessely S Fitzpatrick M. Should psychiatric hospitals completely ban smoking? BMJ. (2015) 351:h5654. 10.1136/bmj.h5654 26536887

[5] Thomas M Richmond R. Smoke-free mental health inpatient facility policies in Australia: variation across states and territories. Aust N Z J Public Health. (2017) 41:329–32. 10.1111/1753-6405.12649 28245510

[6] Ortiz G Schacht L Smoking ban implementation in psychiatric inpatient hospitals: update and opportunity for performance improvement. J Family Med. (2015) 2:1039.

[7] Sohal H Huddlestone L Ratschen E. Preparing for completely smoke-free mental health settings: findings on patient smoking, resources spent facilitating smoking breaks, and the role of smoking in reported incidents from a Large Mental Health Trust in England. Int J Environ Res Public Health. (2016) 13:E256. 10.3390/ijerph13030256 26927143 PMC4808919

[8] Wye P Bowman J Wiggers J Baker A Knight J Carr V. Total smoking bans in psychiatric inpatient services: a survey of perceived benefits, barriers and support among staff. BMC Public Health. (2010) 10:372. 10.1186/1471-2458-10-372 20576163 PMC3091547

[9] Williams JM. Eliminating tobacco use in mental health facilities: patients' rights, public health, and policy issues. JAMA. (2008) 299:571–3. 10.1001/jama.299.5.571 18252888

[10] Hackett M $$Smoke-free state psychiatric facility grounds: is legislation necessary and appropriate to remove tobacco from these treatment settings? New York Law, Law Review (2008/09) 5399–131.

[11] Sullivan D Rees M Medical issues: smoking bans in secure psychiatric hospitals and prisons. J Law Med. (2014) 22:22–30. 25341317

[12] Ratschen E McNeill A Doody GA Britton J. Smoking, mental health, and human rights: a UK judgment. Lancet. (2008) 371:2067–8. 10.1016/S0140-6736(08)60898-3 18572066

[13] Frazer K McHugh J Callinan JE Kelleher C. Impact of institutional smoking bans on reducing harms and secondhand smoke exposure. Cochr Database Syst Rev. (2016) CD011856. 10.1002/14651858.CD011856.pub2 27230795 PMC10164285

[14] Brose LS Simonavicius E McNeill A. Maintaining abstinence from smoking after a period of enforced abstinence – systematic review, meta-analysis and analysis of behaviour change techniques with a focus on mental health. Psychol Med. (2017) 48:669–78. 10.1017/S0033291717002021 28780913 PMC5681216

[15] Kass NE. An ethics framework for public health. Am J Public Health. (2001) 91:1776–82. 11684600 10.2105/ajph.91.11.1776 PMC1446875

[16] Warner J Smoking, stigma and human rights in mental health: going up in smoke? Soc Policy Soc. (2009) 8:275–86. 10.1017/S1474746408004788

[17] Thomas M Richmond R. Addressing the arguments against implementation of smoke-free policies in psychiatric facilities. J Psychiatr Mental Health Nurs. (2017) 24:322–31. 10.1111/jpm.12383 28261996

[18] Lawn S Condon J. Psychiatric nurses' ethical stance on cigarette smoking by patients: determinants and dilemmas in their role in supporting cessation. Int J Mental Health Nurs. (2006) 15:111–8. 10.1111/j.1447-0349.2006.00410.x 16643346

[19] Stubbs J Haw C Garner L Survey of staff attitudes to smoking in a large psychiatric hospital. Psychiatr Bull. (2004) 28:204–7. 10.1192/pb.28.6.204

[20] Khantzian EJ. The self-medication hypothesis of addictive disorders: focus on heroin and cocaine dependence. Am J Psychiatry. (1985) 142:1259–64. 10.1176/ajp.142.11.1259 3904487

[21] Shattell M Andes M. Smoking bans in acute care psychiatric settings: a machiavellian smoke screen? Issues Mental Health Nurs. (2008) 29:201–3. 10.1080/01612840701792274 18293226

[22] Kinsinger FS. Beneficence and the professional's moral imperative. J Chiropractic Hum. (2010) 16:44–6. 10.1016/j.echu.2010.02.006 22693466 PMC3342811

[23] Prochaska JJ Das S Young-Wolff KC. Smoking, Mental Illness, and Public Health. Ann Rev Public Health. (2017) 38:165–85. 10.1146/annurev-publhealth-031816-044618 27992725 PMC5788573

[24] Punnoose S Belgamwar MR Nicotine for schizophrenia. Cochr Database Syst Rev. (2006) 1:CD004838 10.1002/14651858.CD004838.pub2 PMC11807614 16437497

[25] Jochelson K Majrowski B Clearing The Air: Debating Smoke-Free Policies In Psychiatric Units. London: The King's Fund (2006).

[26] Green MA Hawranik PG. Smoke-free policies in the psychiatric population on the ward and beyond: a discussion paper. Int J Nurs Stud. (2008) 45:1543–9. 10.1016/j.ijnurstu.2007.12.004 18304553

[27] Ker S Owens D. Admission to a psychiatric unit and changes in tobacco smoking. Clin Prac Epidemiol Mental Health. (2008) 4:12. 10.1186/1745-0179-4-12 18460210 PMC2397403

[28] Steele R Henderson P Lennon F Swinden D Boredom among psychiatric in-patients: does it matter? Adv Psychiatr Treat. (2013) 19:259–67. 10.1192/apt.bp.112.010363

[29] Steele R Linsley K Relieving in-patient boredom in general hospitals: the evidence for intervention and practical ideas. BJPsych Adv. (2015) 21:63–70. 10.1192/apt.bp.113.011908

[30] Cavazos-Rehg PA Breslau N Hatsukami D Krauss MJ Spitznagel EL Grucza RA. Smoking cessation is associated with lower rates of mood/anxiety and alcohol use disorders. Psychol Med. (2014) 44:2523–35. 10.1017/S0033291713003206 25055171 PMC4122254

[31] Hollen V Ortiz G Schacht L Mojarrad MG Lane GM Jr Parks JJ. Effects of adopting a smoke-free policy in state psychiatric hospitals. Psychiatr Serv. (2010) 61:899–904. 10.1176/ps.2010.61.9.899 20810588

[32] Robson D Spaducci G McNeill A Stewart D Craig TJK Yates M. Effect of implementation of a smoke-free policy on physical violence in a psychiatric inpatient setting: an interrupted time series analysis. Lancet Psychiatry. (2017) 4:540–6. 10.1016/S2215-0366(17)30209-2 28624180

[33] National Mental Health Consumer and Carer Forum Advocacy Brief: Smoking and Mental Health. (2014). Available online at: http://nmhccf.org.au/publication/smoking-and-mental-health

[34] Prochaska JJ. Failure to treat tobacco use in mental health and addiction treatment settings: a form of harm reduction? Drug Alcohol Depend. (2010) 110:177–82. 10.1016/j.drugalcdep.2010.03.002 20378281 PMC2916693

[35] Prochaska JJ Gill P Hall SM. Treatment of tobacco use in an inpatient psychiatric setting. Psychiatr Serv. (2004) 55:1265–70. 10.1176/appi.ps.55.11.1265 15534015

[36] George O Koob GF Chapter 1 - Overview of nicotine withdrawal and negative reinforcement (Preclinical). In: Hall FS Young JW Der-Avakian A, Editors. Negative Affective States and Cognitive Impairments in Nicotine Dependence. San Diego, CA: Academic Press (2017), p. 1–20.

[37] Taylor G McNeill A Girling A Farley A Lindson-Hawley N Aveyard P. Change in mental health after smoking cessation: systematic review and meta-analysis. BMJ. (2014) 348:g1151. 10.1136/bmj.g1151 24524926 PMC3923980

[38] Morris CD Waxmonsky JA May MG Tinkelman DG Dickinson M Giese AA. Smoking reduction for persons with mental illnesses: 6-month results from community-based interventions. Commun Mental Health J. (2011) 47:694–702. 10.1007/s10597–011-9411-z 21556784

[39] Moritsugu KP. The 2006 report of the surgeon general: the health consequences of involuntary exposure to tobacco smoke. Am J Prevent Med. (2007) 32:542–3. 10.1016/j.amepre.2007.02.026 17533072

[40] Willemsen MC Görts CA Van Soelen P Jonkers R Hilberink SR. Exposure to environmental tobacco smoke (ETS) and determinants of support for complete smoking bans in psychiatric settings. Tobacco Control. (2004) 13:180–5. 10.1136/tc.2003.004804 15175537 PMC1747858

[41] Ballbe M Sureda X Martinez-Sanchez JM Salto E Gual A Fernandez E. Second-hand smoke in mental healthcare settings: time to implement total smoke-free bans? Int J of Epidemiol. (2013) 42:886–93. 10.1093/ije/dyt014 23543600

